# ﻿A new species of *Melinnopsis* (Annelida, Melinnidae) from the Porcupine Abyssal Plain, northeast Atlantic

**DOI:** 10.3897/zookeys.1265.171206

**Published:** 2025-12-23

**Authors:** Laetitia M. Gunton, Amanda Serpell-Stevens, Kashaf Riaz, Tammy Horton

**Affiliations:** 1 School of Ocean and Earth Science, University of Southampton, Southampton, SO14 3ZH, UK University of Southampton Southampton United Kingdom; 2 National Oceanography Centre Southampton, Southampton SO14 3ZH, UK National Oceanography Centre Southampton Southampton United Kingdom; 3 School of the Environment and Life Sciences, University of Portsmouth, King Henry Building, Portsmouth PO1 2DY, UK University of Portsmouth Portsmouth United Kingdom

**Keywords:** Atlantic Ocean, deep sea, molecular, phylogenetics, polychaete, taxonomy

## Abstract

A new species of polychaete worm, *Melinnopsis nathanieli***sp. nov.** (Annelida, Melinnidae), is described from the Porcupine Abyssal Plain in the northeast Atlantic at ~4850 m depth. Forty-two specimens were collected during RRS *James Cook* cruises JC247 and JC263 in 2023 and 2024 respectively. The new species is morphologically similar to *Melinnopsis gardelli* and *Melinnopsis chadwicki*, described from deep Australian waters, but differs from these species in having fewer (~ 16) abdominal uncini in a row compared with 30 in both *M. gardelli* and *M. chadwicki*. Phylogenetic relationships among the new species and other species in the family Melinnidae were assessed using the nuclear 18S, and the mitochondrial 16S and cytochrome oxidase subunit I (COI) gene fragments. The new species is genetically distinct from all other species of *Melinnopsis*, where molecular data is available, and genetically most similar to *M. gardelli* (pairwise distance 3.5%). Tubes of *M. nathanieli***sp. nov.** were observed to act as a biogenic substrate for Actiniaria and Ascidiacea. Seafloor images of *Melinnopsis nathanieli***sp. inc.** at the Porcupine Abyssal Plain show the worm’s tube positioned perpendicular to the sediment surface, and the long buccal tentacle protruding from the tube. A table containing key characters of all known *Melinnopsis* species is presented. This is the first species of *Melinnopsis* to be described from the northeast Atlantic.

## ﻿Introduction

Melinnidae Chamberlin, 1919 is a family of tubicolous worms, commonly known as ‘grapple worms’ due to the presence of stout recurved hooks, resembling grappling hooks, which are present in some species. Species of the family Melinnidae are generally restricted to deeper waters. The family currently contains 52 accepted species (Rouse et al. 2022; Read and Fauchald 2025a). Traditionally, Melinninae Chamberlin, 1919 and Ampharetinae Malmgren, 1866 were considered subfamilies, within the family Ampharetidae Malmgren, 1866. Recently, Stiller et al. (2020) used molecular and morphological data to show that Melinninae, was more closely affiliated to the Terebellidae Johnston, 1846, rather than being a sister group to Ampharetinae, and thus assigned family status to Melinnidae. Currently, the family Melinnidae contains four genera, *Isolda* Müller, 1858, *Melinna* Malmgren, 1866, *Melinnopsides* Day, 1964 and *Melinnopsis* McIntosh, 1885.

Within the genus *Melinnopsis*, there are 20 recognised species (Read and Fauchald 2025b). The genus was erected for *Melinnopsis atlantica* McIntosh, 1885 collected off Chesapeake Bay, Maryland in the northwest Atlantic Ocean at ~ 3100 m (1700 fathoms) during the HMS *Challenger* expedition. Due to the brevity of the original description and lack of justification for the new genus, the ensuing taxonomic history has been complicated, as outlined by Gunton et al. (2020). The most recently described species is *Melinnopsis shinkaiae* Jimi, Hookabe, Woo & Fujiwara, 2025 from the Daiichi-Kashima Seamount off Japan in the northwest Pacific Ocean at 3623 m depth. *Melinnopsis shinkaiae* was described based on two specimens using only morphological characters, as attempts to extract and amplify DNA were unsuccessful (Jimi et al. 2025). The most recent descriptions employing genetic data are those of Gunton et al. (2020) which included specimens from Australian waters in the southwest Pacific Ocean at 1006–2821 m depth. Gunton et al. (2020) used molecular (COI, 16S and 18S gene fragments) and morphological data to describe the genetically distinct but morphologically similar species, *Melinnopsis chadwicki* Gunton, Kupriyanova & Alvestad, 2020 and *Melinnopsis gardelli* Gunton, Kupriyanova & Alvestad, 2020. To date, only two species of *Melinnopsis* have been described from the Atlantic Ocean, *M. atlantica*, the aforementioned type species of the genus, and *Melinnopsis angolensis* Hilbig, 2005 described from the Angola Basin, southeast Atlantic at 5385–5439 m depth.

The Porcupine Abyssal Sustained Observatory (PAP-SO) is a multidisciplinary open-ocean time-series study site in the northeast Atlantic (48°50'N, 16°30'W). PAP-SO has been studied since 1985 and is now the longest running abyssal time-series site in the world (Hartman et al. 2021). Material collected from the site is deposited in The Discovery Collections, National Oceanography Centre, Southampton, an internationally important repository of deep-sea marine benthic and pelagic invertebrate and fish specimens obtained since 1925. Despite this substantial collection of material, the annelid fauna is poorly characterised at the PAP-SO. There have been five ecological studies of macrofauna (including annelids at family level) (Paterson et al. 1994; Glover et al. 2001; Galeron et al. 2001; Soto et al. 2010; Laguionie-Marchais et al. 2013), one study on annelid recruitment (Vanreusel et al. 2001), and four species-level studies on annelids from the PAP-SO (Dauvin et al. 1994; Paterson et al. 1998; Soto-Oyarsun 2008; Laguionie-Marchais 2015). To date, only four annelid species from two families have been described or had distributions recorded from the site including the spionids *Aurospio abranchiata* Neal, Paterson & Soto in Paterson et al., 2016 (type locality Portuguese margin, distribution including PAP at 4800 m), *Aurospio tribranchiata* Paterson & Soto in Paterson et al., 2016 (type locality PAP at 4800 m), *Aurospio dibranchia* Maciolek, 1981 (type locality Argentine Basin southwest Atlantic, distribution including near PAP off Ireland, northeast Atlantic at 1500-3350 m), and a pilargid, *Sigambra magnuncus* Paterson & Glover, 2000 (type locality PAP ca 4850 m). Thus, there is a clear lack of detailed knowledge on the polychaete fauna from the PAP-SO.

The present study describes a new species of *Melinnopsis*, from depths of 4850 m at the Porcupine Abyssal Plain (PAP), northeast Atlantic Ocean. The phylogenetic position of the new species is assessed within the genus *Melinnopsis*. Furthermore, in-situ seafloor images of *Melinnopsis* at PAP-SO are presented.

## ﻿Materials and methods

### ﻿Sample collection

**Figure 1.**
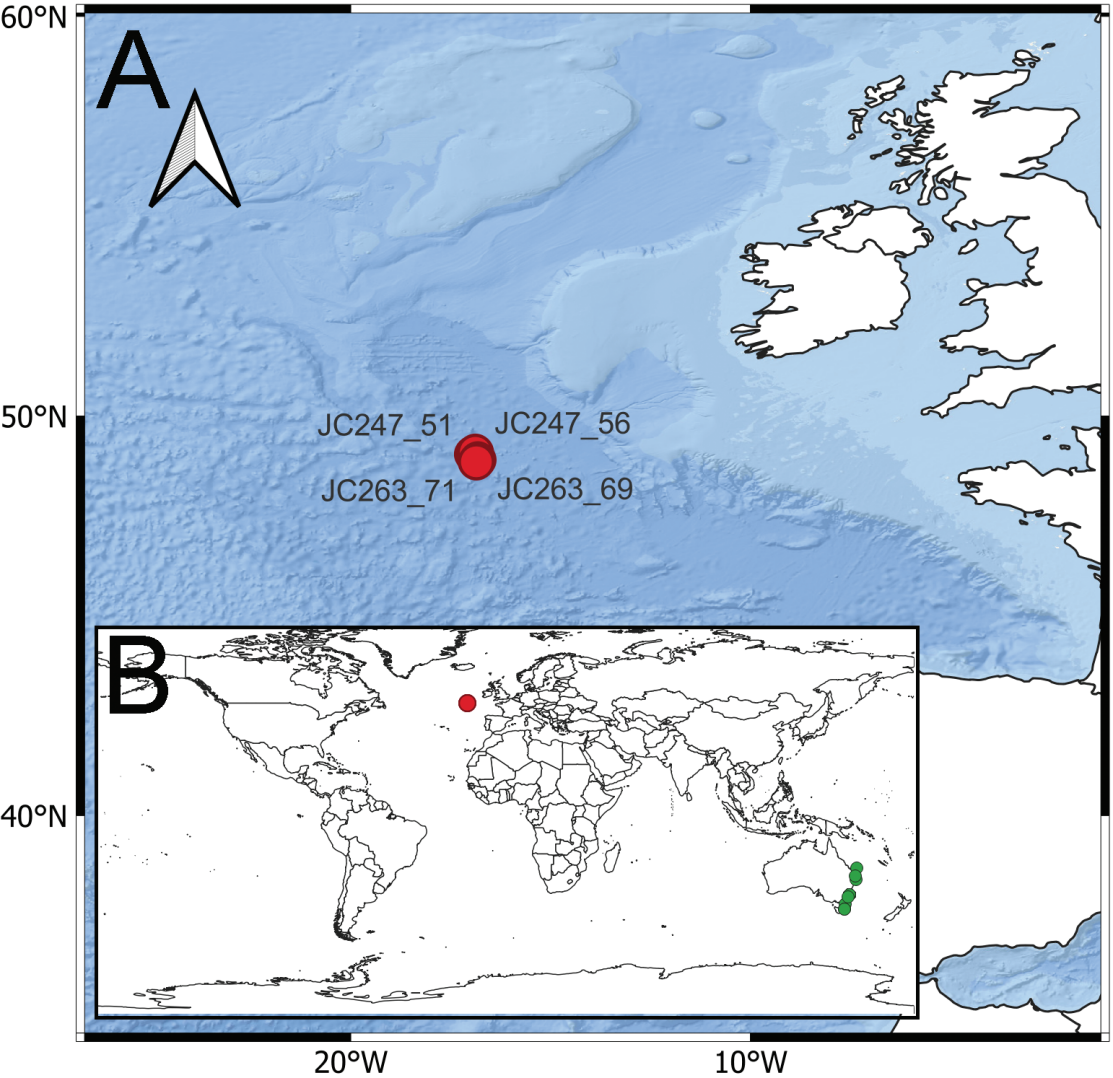
**A.** Map of station locations at Porcupine Abyssal Plain Sustained Observatory in Northeast Atlantic. Red dots indicate sampling sites; **B.** Global distribution of *Melinnopsis nathanieli* sp. nov. (red dots) and sister species *M. gardelli* (green dots).

Samples were collected during two RRS James Cook cruises to the PAP-SO; JC247 and JC263, in 2023 and 2024 respectively (Table 1, Fig. 1). Onboard, the OTSB14 (semi-balloon otter trawl) was utilised. The resulting trawl samples were washed in filtered sea water, and the annelids picked out. Annelid tubes were sorted either separately or with associated taxa, various Actiniaria and ascidians. All specimens were fixed in 100% ethanol.

**Table 1.** RRS *James Cook* cruise and trawl station locations for *Melinnopsis nathanieli* sp. nov. specimens collected.

Cruise	Station No.	Date	Depth (m)	Latitude N start, Longitude W start	Latitude N finish, Longitude W finish
JC247	51	16/05/23–17/05/23	4844–4846	49°02.63’N, 016°56.95’W	48°58.10’N, 016°57.81’W
JC247	56	17/05/23–18/05/23	4843–4848	49°05.43’N, 016°53.02’W	49°01.12’N, 016°57.87’W
JC263	69	06/06/24–07/06/24	4835–4836	48°54.03’N, 016°49.26’W	49°00.70’N, 016°50.22’W
JC263	71	07/06/24–08/06/24	4831–4838	48°51.21’N, 016°51.45’W	48°57.45’N, 016°51.85’W

### ﻿Morphological investigations

Identification was performed in the laboratory at the National Oceanography Centre, Southampton, where specimens were transferred to 80% ethanol. Worms were carefully extracted from the tubes using forceps, length and width measurements were taken. Identification was made using a stereo microscope, Leica M165C. Light photographs of specimens were taken using a Leica Flexacam C5 camera attached to the stereo microscope.

Paratypes of *Melinnopsis nathanieli* sp. nov. were dehydrated in ethanol, critical point dried, coated in 80:20 gold: palladium mix 15–20 nm, and examined under the Scanning Electron Microscope Tescan Mira 3 FEG-SEM at the University of Portsmouth Electron Microscopy and Microanalysis Unit. Type material is lodged at the Natural History Museum in London, with **NHMUK** registration numbers, all other specimens are retained at The Discovery Collections, National Oceanography Centre, Southampton (**DISCOLL**). Other material included in this study includes the holotype of *Melinnopsis atlantica* (BMNH 1885.12.1.330) from the Natural History Museum, London.

### ﻿DNA extraction, amplification, and sequencing

Molecular analysis was performed at Portsmouth University. Tissue samples were collected from seven specimens. DNA extraction was performed using a QIAGEN DNeasy® Blood & Tissue Kit following the manufacture’s protocols. PCR amplification of the COI, 16S and 18S genes was conducted using ten sets of primers (Table 2). Polymerase chain reaction (PCR) mixtures consisted of 12.5 µl of Thermo Scientific™ DreamTaq PCR Master Mix, 2 µl of each primer (forward and reverse), 7.5 µl of nuclease-free water and 1 µl of template DNA, making a total mixture of 25 µl.

**Table 2.** Primers used for PCR and Sanger sequencing.

Gene	Primer	Sequence 5’-3’	Direction	Reference
16S	Ann16SF	GCGGTATCCTGACCGTRCWAAGGTA	Forward	Sjölin et al. (2005)
	16SbrH	CCGGTCTGAACTCAGATCACGT	Reverse	Palumbi (1991)
18S	18e	CTGGTTGATCCTGCCAGT	Forward	Hillis and Dixon (1991)
	18L	GAATTACCGCGGCTGCTGGCACC	Reverse	Halanych et al. (1995)
	18F509	CCCCGTAATTGGAATGAGTACA	Forward	Struck et al. (2002)
	18R	GTCCCCTTCCGCAATTYCTTTAAG	Reverse	Passamaneck et al. (2004)
	TimA	AMC TGG TTG ATC CTG CCA G	Forward	Norén and Jondelius (1999)
	1100R2modified	CGG TAT CTG ATC GTC TTC GA	Reverse	Kupriyanova et al. (2006)
COI	polyLCO	GAYTATWTTCAACAAATCATAAAGATATTGG	Forward	Carr et al. (2011)
	polyHCO	TAMACTTCWGGGTGACCAAARAATCA	Reverse	Carr et al. (2011)

PCRs were conducted in a Thermal Cycler with the following conditions; COI: 94 °C/1 min, 5 cycles 94°/40 s, 45°/40 s, 72°/60 s, followed by 35 cycles 94°/40 s, 51°/40 s, 72°/60 s, and finally 72°/5 min. 16S: 94°/4 min, 35 cycles of 94°/30 s, 48°/30 s, 72°/60 s, and finally 72°/8 min. 18S (TimA/1100R2): 94 °C/3 min, 40 cycles 94 °C/ 30 s, 52 °C/ 30 s, 72 °C/ 30 s and finally 72°/5 min. 18S (18E/18L and 18F509/18R): 94 °C/3 min, 35 cycles 94 °C/ 60 s, 42 °C/ 90 s, 72 °C/ 120 s and finally 72°/7 min.

The quantity of PCR products was detected using gel electrophoresis and visualised using a Gel Documentation System. Successful PCR products were sent to GENEWIZ (https://www.genewiz.com/en-gb/) where they were purified, and standard Sanger sequencing was performed.

### ﻿Sequence analysis

Overlapping fragments were assembled into consensus sequences and edited in Geneious Prime 2019.0.4 (https://www.geneious.com). A BLAST analysis (Altschul et al. 1990) was performed to confirm the correct region had been amplified, to compare with other sequences on GenBank, and to check for contamination. New sequences were submitted to GenBank (Table 3). Additional sequences from the genus *Melinnopsis* (4 species and 24 sequences), and one species of *Melinna*, selected as an outgroup, were downloaded from GenBank (Table 3).

**Table 3.** Melinnidae taxa used in molecular phylogenetic analysis with museum voucher number, sampling location, depth, GenBank accession numbers. Institutional abbreviations: RUB - Ruhr-Universitat Bochum, SIO-BIC - Scripps Institution of Oceanography Benthic Invertebrate Collection, ZMBN - Department of Natural History, University Museum of Bergen, AM - Australian Museum, NHMUK- Natural History Museum, London. Dashes (—) indicate no data available.

Taxon	Voucher	Collection location	Depth (m)	GenBank or BOLD accession number			Publication
				COI	16S	18S	
*** Melinnopsis ***							
Melinnopsis cf. armipotens (Moore, 1923)	SIO:BIC:A12604	Costa Rica: Pacific Ocean, Subduction Plume	3502	PQ449274	—	—	Seid et al. 2025
*Melinnopsis* sp.	RUB Msp_01	Antarctica	2057	RUMS096-09	—	—	Unpublished
*Melinnopsis* sp.	RUB Msp_09	Antarctica	2057	RUMS104-09	—	—	Unpublished
*Melinnopsis* sp.	RUB Msp_27	Antarctica	2057	RUMS122-09	—	—	Unpublished
*Melinnopsis* sp.	RUB Msp_28	Antarctica	2057	RUMS123-09	—	—	Unpublished
*Melinnopsis* sp.	RUB Msp_29	Antarctica	2057	RUMS124-09	—	—	Unpublished
*Melinnopsis chadwicki* Gunton et al. 2020	AM W.50414	off Moreton Bay, Australia	1071-1138	MT556172	MT556641	MT561568	Gunton et al. 2020
*Melinnopsis chadwicki* Gunton et al. 2020	AM W.52949	Coral Sea Marine Park, Australia	1013-1093	MT556174	MT556643	MT561570	Gunton et al. 2020
*Melinnopsis chadwicki* Gunton et al. 2020	AM W.52948	Coral Sea Marine Park, Australia	1013-1093	MT556173	MT556642	MT561569	Gunton et al. 2020
*Melinnopsis gardelli* Gunton et al. 2020	AM W.52539	Jervis Marine Park, Australia	2650-2636	MT556177	MT556646	MT561573	Gunton et al. 2020
*Melinnopsis gardelli* Gunton et al. 2020	AM W.50735	Jervis Marine Park, Australia	2650-2636	MT556175	MT556644	MT561571	Gunton et al. 2020
*Melinnopsis gardelli* Gunton et al. 2020	AM W.51476	Freycinet Marine Park, Australia	2820-2751	MT556176	MT556645	MT561572	Gunton et al. 2020
*Melinnopsis nathanieli* sp. nov. (holotype)	NHMUK ANEA 2025.3262	PAP NE Atlantic	4843-4848	PX149846	PX169426	—	This study
*Melinnopsis nathanieli* sp. nov.	DISCOLL-JC247-056-POLY-006	PAP NE Atlantic	4843-4848	PX149845	PX169425	PX169420	This study
*Melinnopsis nathanieli* sp. nov.	DISCOLL-JC247-056-POLY-009	PAP NE Atlantic	4843-4848	PX149844	PX169424	PX169419	This study
*Melinnopsis nathanieli* sp. nov.	DISCOLL-JC247-056-POLY-011	PAP NE Atlantic	4843-4848	PX149843	PX169423	PX169418	This study
*Melinnopsis nathanieli* sp. nov.	DISCOLL-JC247-056-POLY-013	PAP NE Atlantic	4843-4848	PX149842	—	PX169417	This study
*Melinnopsis nathanieli* sp. nov.	DISCOLL-JC247-056-POLY-015	PAP NE Atlantic	4843-4848	PX149841	PX169422	PX169416	This study
*Melinnopsis nathanieli* sp. nov.	DISCOLL-JC263-071-POLY-024	PAP NE Atlantic	~4850	—	PX169421	—	This study
**Outgroup *Melinna***							
*Melinna cristata* (M. Sars, 1851)	ZMBN 95306	Skagerrak, Norway	212	MG270118	MG253102	MG253147	Eilertsen et al. 2017

Sequences were aligned using the Geneious plugins with the default settings: MAFFT (Katoh et al. 2002) for 16S and 18S and MUSCLE (Edgar 2004) for COI. Pairwise genetic distances for COI and 16S were calculated in Mega v. 11.0.13 (Tamura et al. 2021). Concatenated sequences for all three genes were made in Geneious. JModelTest (Darriba et al. 2012) was used to find the best model using the Akaike Information Criterion (AIC). The model GTR+I+G was selected for COI, TIM2+I for 16S and TrN for 18S. Phylogenetic trees were constructed using IQ-TREE version 2 (Minh et al. 2020) and run for 10000 bootstrap replicates. Trees were visualised in FigTree v. 1.4.4 (Rambaut 2018) and edited in Adobe Illustrator.

## ﻿Results

### ﻿Molecular results

**Figure 2.**
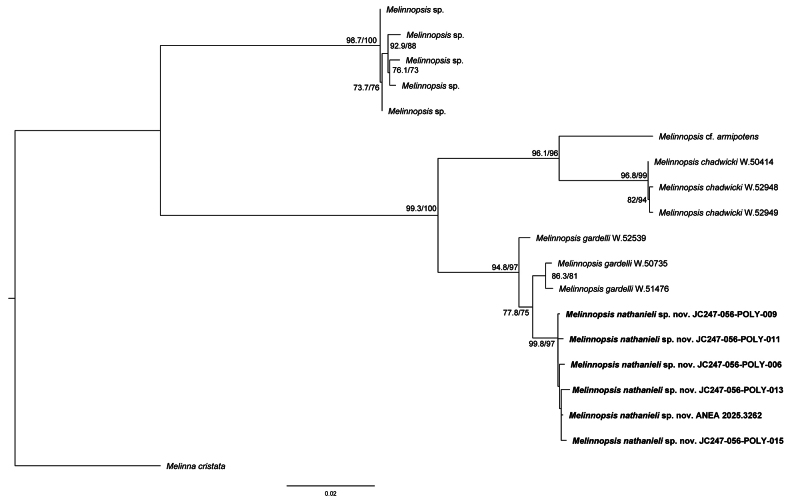
Maximum likelihood tree of *Melinnopsis* from IQTREE based on COI, 16S, and 18S gene fragments. Scale bar represents 0.02 substitutions per site.

The maximum likelihood analysis of *Melinnopsis* for three concatenated gene fragments (COI, 16S and 18S) recovered a tree with two well-supported (SH-aLRT: 100%, UFBoot: 100%) clades within *Melinnopsis* (Fig. 2). The first well-supported clade (SH-aLRT: 98.7%, UFBoot: 100%) contained specimens identified as *Melinnopsis* sp. from Antarctica. The second clade (SH-aLRT: 99.3%, UFBoot: 100%), contained Melinnopsis cf. armipotens, *M. chadwicki*, *M. gardelli* and *M. nathanieli* sp. nov. Specimens attributed to *M. nathanieli* sp. nov. formed a well-supported monophyletic group (SH-aLRT: 99.8%, UFBoot: 97%), these were sister to a clade containing two specimens of *M. gardelli* (AM W.20735 and AM W.51476) recovered with poor support (SH-aLRT: 77.8%, UFBoot: 75%). Sister to the clade containing *M. nathanieli* sp. nov. and *M. gardelli*, one specimen of *M. gardelli* (AM W.52539) was recovered.

The COI intraspecific pairwise genetic distances within *M. nathanieli* sp. nov. ranged 0.006–0.015. The single closest COI sequence of *M. nathanieli* sp. nov. was *M. gardelli* AM W.50735 sequence (0.035 difference).

### ﻿Taxonomy

#### ﻿Order Terebellida

**Suborder Terebelliformia**

**Family Melinnidae Chamberlin, 1919**

##### ﻿ Melinnopsis

Taxon classificationAnimaliaAnnelidaMelinnidae

McIntosh, 1885

4414FDCB-02A3-515D-9877-ED37DBE3134E

Melinnopsis McIntosh, 1885 (including Amelinna Hartman, 1969; Melinnexis Annenkova, 1931; and Melinnides Wesenberg-Lund, 1950) sensu Reuscher et al. (2015).

###### Type species.

*Melinnopsis atlantica* McIntosh, 1885 (type lodged at the Natural History Museum in London U.K., catalogue number 1885.12.1.330). Type locality off Chesapeake Bay, NW Atlantic, 3109 m.

###### Generic diagnosis.

Large buccal tentacles occurring with smaller ones. Four pairs of branchiae. Post-branchial hooks absent. Brittle acicular neurochaetae in segments II–IV or II–V. Twelve to 14 thoracic uncinigers. Uncini with subrostral process.

###### Remarks.

The new species described here conforms to the generic diagnosis of Reuscher et al. (2015), in possessing one long buccal tentacle, four pairs of branchiae, acicular chaetae on segments II-V, 12 or 13 thoracic uncinigers, uncini with subrostral process and lacking post-branchial hooks (dorsal hooks).

##### ﻿ Melinnopsis nathanieli sp. nov.

Taxon classificationAnimaliaAnnelidaMelinnidae

510576F5-F371-529C-9ECB-11F144D84A1B

https://zoobank.org/A0D97042-8E76-4DEC-8103-5602CCDB688B

###### Type material.

***Holotype***: • One specimen; NHMUK ANEA 2025.3262, incomplete, end of abdomen missing; Atlantic, Porcupine Abyssal Plain Sustained Observatory; OTSB14; Start 49°05.43'N, 016°53.02'W, End 49°01.12'N, 016°57.87'W; depth 4843–4848 m; 17/05/23–18/05/23; RRS James Cook Cruise 247, Station 056; COIPX149846, 16S PX169426. ***Paratypes***: Total six specimens. • NHMUK ANEA 2025.3263; NHMUK ANEA 2025.3264, complete specimen; Atlantic, Porcupine Abyssal Sustained Observatory; OTSB14; Start 48°51.21'N, 016°51.45'W, End 48°57.45'N, 016°51.85'W; depth 4831–4838 m; 07/06/24–08/06/24; RRS James Cook Cruise 263, Station 071. • NHMUK ANEA 2025.3265, buccal tentacle extended 10 mm; NHMUK ANEA 2025.3266; NHMUK ANEA 2025.3267 (SEM specimen); NHMUK ANEA 2025.3268 (SEM specimen), complete specimen broken in two fragments; Atlantic, Porcupine Abyssal Plain Sustained Observatory; OTSB14; Start 48°54.03'N, 016°49.26'W, End 49°00.70'N, 016°50.22'W; depth 4835–4836 m; 06/06/24–07/06/24; RRS James Cook Cruise 263, Station 069.

###### Other material examined.

Total 35 specimens. • DISCOLL-JC247-056-POLY-003; DISCOLL-JC247-056-POLY-004; DISCOLL-JC247-056-POLY-005; DISCOLL-JC247-056-POLY-006 (COIPX149845; 16S PX169425; 18S PX169420); DISCOLL-JC247-056-POLY-007; DISCOLL-JC247-056-POLY-008; DISCOLL-JC247-056-POLY-009 (COIPX149844; 16S PX169424; 18S PX169419); DISCOLL-JC247-056-POLY-010; DISCOLL-JC247-056-POLY-011 (COIPX149843; 16S PX169423; 18S PX169418); DISCOLL-JC247-056-POLY-012; DISCOLL-JC247-056-POLY-013 (COIPX149842; 18S PX169417); DISCOLL-JC247-056-POLY-014; DISCOLL-JC247-056-POLY-015 (COIPX149841; 16S PX169422; 18S PX169416); DISCOLL-JC247-056-POLY-016; DISCOLL-JC247-056-POLY-018; DISCOLL-JC247-056-POLY-019; DISCOLL-JC247-056-POLY-020; DISCOLL-JC247-056-POLY-021; DISCOLL-JC247-056-POLY-022; DISCOLL-JC247-056-POLY-023; DISCOLL-JC247-056-POLY-024; DISCOLL-JC247-056-POLY-025; all same collection data as for holotype. • DISCOLL-JC247-051-POLY-039; Atlantic, Porcupine Abyssal Plain Sustained Observatory; OTSB14; Start 49°02.63'N, 016°56.95'W, End 48°58.10'N, 016°57.81'W; depth 4844–4846 m; 16/05/23-15/05/23; RRS James Cook Cruise 247, Station 051. • DISCOLL-JC263-069-POLY-033; DISCOLL-JC263-069-POLY-034; DISCOLL-JC263-069-POLY-035; DISCOLL-JC263-069-POLY-036; DISCOLL-JC263-069-POLY-037; Atlantic, Porcupine Abyssal Plain Sustained Observatory; OTSB14; Start 48°54.03'N, 016°49.26'W, End 49°00.70'N, 016°50.22'W; depth 4835–4836 m; 06/06/24–07/06/24; RRS James Cook Cruise 263, Station 069. • DISCOLL-JC263-071-POLY-023, complete specimen (5 branchiae); DISCOLL-JC263-071-POLY-024 (16S PX16942); DISCOLL-JC263-071-POLY-025, 2 fragments; DISCOLL-JC263-071-POLY-027; DISCOLL-JC263-071-POLY-028; DISCOLL-JC263-071-POLY-029; DISCOLL-JC263-071-POLY-030; Atlantic, Porcupine Abyssal Plain Sustained Observatory; OTSB14; Start 48°51.21'N, 016°51.45'W, End 48°57.45'N, 016°51.85'W; depth 4831–4838 m; 07/06/24–08/06/24; RRS James Cook Cruise 263, Station 071.

###### Additional comparative material.

BMNH 1885.12.1.330, holotype of *Melinnopsis atlantica* McIntosh, 1885, off Chesapeake Bay, North America, 37°25.002'N, 71°40.002'W, HMS Challenger, Stn. 44, 1700 fathoms (3109 m), 02/05/1873, 2 short anterior fragments, 1 posterior fragment and ~ 9 tube fragments.

###### Description.

**Figure 3.**
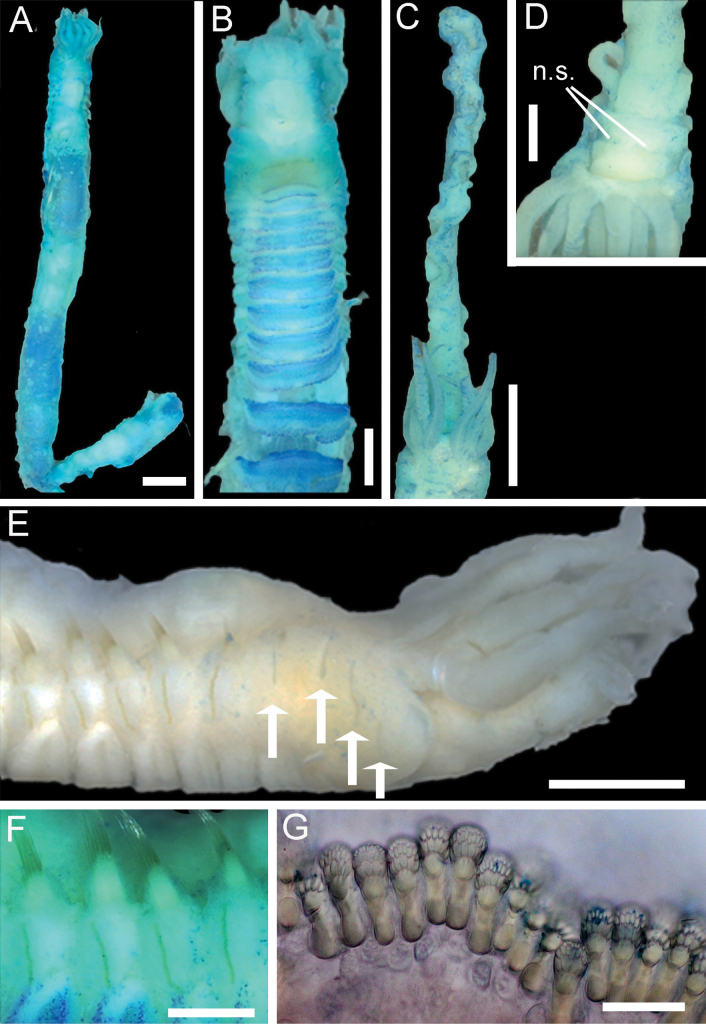
Light images of *Melinnopsis nathanieli* sp. nov. **A.** Holotype NHMUK ANEA 2025.3262 entire body dorsal view; **B.**NHMUK ANEA 2025.3262 anterior section ventral view; **C.**NHMUK ANEA 2025.3265 prostomium and long buccal tentacle; **D.**NHMUK ANEA 2025.3265 prostomium; **E.**NHMUK ANEA 2025.3262 anterior body lateral view, arrows indicate acicular neurochaetae; **F.**NHMUK ANEA 2025.3262 thoracic chaetigers; **G.**NHMUK ANEA 2025.3262 abdominal uncini. Abbreviations: n.s. = nuchal slits. Scale bars: 2 mm (**A**); 1 mm (**B, D, E**); 3 mm (**C**); 0.5 mm (**F**); 20 µm (**G**).

(based on holotype NHMUK ANEA 2025.3262) Holotype 35 mm length for more than 25 chaetigers, widest at post-branchial region 2 mm, thereafter gradually tapering to abdomen (1 mm width) (Fig. 3A). Thorax with 16 or 17 chaetigers (lower thorax damaged so exact number of chaetigers uncertain). Neurochaetae as small acicular spines on segments II–V and uncini on remaining > 21 chaetigers.

Prostomium with well-defined anterior and posterior sections separated by a pair of deep transverse nuchal slits meeting mid-dorsally (Fig. 3D). Anterior part of prostomium whole, without any distinct lobes, and with a slightly raised lip. No eyespots or pigmented glandular bands present. No speckled pigment on anterior part of prostomium. Segment I continued ventrally forming lower margin of mouth with no crenulations on the ventral side (Fig. 3B).

Buccal tentacles in holotype missing, only one large stump remaining.

**Figure 4.**
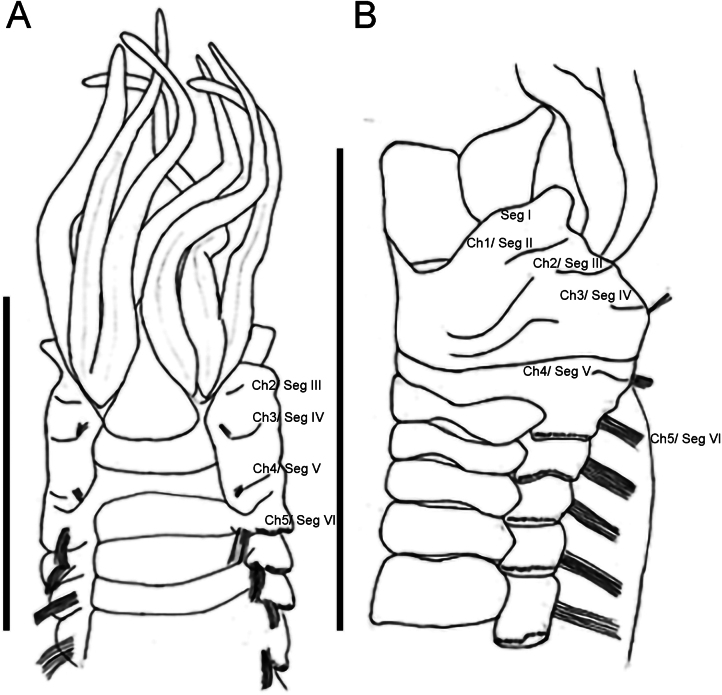
Line drawings of paratype NHMUK ANEA 2025.3264 *Melinnopsis nathanieli* sp. nov. **A.** Anterior dorsal view; **B.** Anterior lateral view. Abbreviations: Ch = chaetiger, Seg = segment. Scale bars: 4 mm (**A**); 5 mm (**B**).

Lateral wings of anterior body between prostomium and segment V slightly arched, peak approx. segment IV (Figs 3E, 4B).

**Figure 5.**
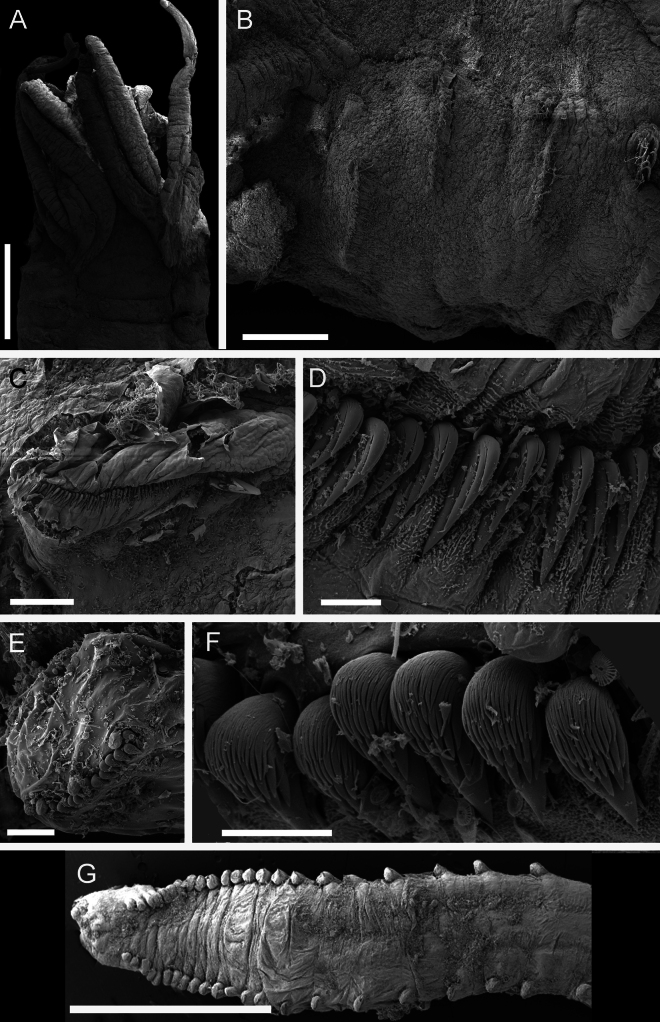
Scanning electron microscope images of *Melinnopsis nathanieli* sp. nov. paratypes. **A.**NHMUK ANEA 2025.3267 Anterior dorsal view; **B.**NHMUK ANEA 2025.3267 acicular chaetae; **C.**NHMUK ANEA 2025.3268 thoracic uncinigers; **D.**NHMUK ANEA 2025.3268 thoracic uncini; **E.**NHMUK ANEA 2025.3268 abdominal unciniger; **F.**NHMUK ANEA 2025.3268 abdominal uncini; **G.**NHMUK ANEA 2025.3268 pygidium dorsal view. Scale bars: 1 mm (**A**); 500 µm (**B**); 100 µm (**C**); 10 µm (**D, F**); 50 µm (**E**); 2 mm (**G**).

Segment I collar-like, laterally and ventrally encompassing head region. Branchiae emerging together on dorsal branchial ridge at level of segment II, arranged in two basally fused groups of four, three branchiae in front and one situated slightly behind (towards the anterior) (Figs 4A, 5A), the latter being the longest pair. Inner- and anteriormost branchia of each group completely separate, not joined by membrane. Branchiae in cross-section circular to slightly flattened smooth with central groove, gently tapering to filiform tips. Post-branchial dorsal membrane inconspicuous (Fig. 5A).

Post-branchial hooks absent. Segmentation visible dorsally in post-branchial area. No visible nephridial papillae.

Notochaetae from segment IV, neurochaetae from segment II (Figs 4B, 5B).

Capillary notochaetae starting from segment IV present in 12 or 13 thoracic chaetigers (lower thorax damaged exact number of chaetigers uncertain). Chaetiger 3 (segment IV) with few fine notochaetal capillaries and chaetiger 4 (segment V) with more abundant fine notochaeta arising from small slightly projecting notopodia (Fig. 5B). Short cylindrical notopodia with thicker capillaries evident from chaetiger 5. Notochaetae arranged in double rows, those of anterior rows shorter.

Rudimental abdominal notopodia missing. No small, rounded projections, evident in notopodial positions, no cilia observed.

Neurochaetae as small acicular spines with lanceolate tips on segments II–V. Neuropodial uncini from chaetiger 5 (segment VI) (Fig. 5B), present in 12 thoracic uncinigers and abdominal chaetigers. Holotype incomplete with ~ 15 abdominal uncinigers.

Thoracic uncini emerging subdistally on short flaps from chaetigers 5 to approximately chaetiger 11 (Figs 3F, 5C). Abdominal uncini mostly missing in holotype but arranged on narrow lappets (Fig. 5E).

Uncini of thoracic uncinigers with three teeth (two small, one larger) in one vertical row over rostral tooth, subrostral process and basal prow (Figs 3G, 5D).

Thoracic uncini in a single line with ~70 uncini (Fig. 5C).

Abdominal uncini in a single line with ~16 uncini (Fig. 5E).

Pygidium missing in holotype.

**Methyl blue staining pattern.** Use of methyl blue in holotype reveals weak staining of prostomium. Posterior section of prostomium with speckled staining (Fig. 3B). Strong staining transversely on segments I–IV (Fig. 3A). Branchiae very lightly speckled. No clear lateral staining. Strong staining of ~13 or 14 ventral shields, staining strong in anterior section of ventral shield, light staining of posterior section of shield. This pattern is more defined towards the posterior of the worm. Shields do not cover entire ventral surface of the segment, stopping short at the uncini (Fig. 3B).

**Tube.** Missing in holotype.

###### Variations.

Variation in other specimens up to 48 mm length for more than 60 chaetigers, widest at post-branchial region 3 mm, thereafter gradually tapering to abdomen (1 mm width) and pygidium. Thorax with 16 chaetigers; neurochaetae as small acicular spines on segments II–V and uncini on remaining > 56 chaetigers.

One large buccal tentacle (≤15 mm), edges of tentacle undulate in small folds (Fig. 3C). Four smaller tentacles (2 pairs) ridged and smooth measuring ~1 mm length.

Branchiae ~1/5 the length of longest buccal tentacle.

Thirteen thoracic uncini from chaetiger 5–17. Some specimens with ≤ 49 abdominal uncinigers.

Uncini lappets decreasing in size until pygidium, minute at the end. First abdominal neuropodia widely separated, more closely spaced towards the pygidium, last seven neuropodia very close together.

Uncini of abdominal uncinigers with numerous teeth over rostral tooth, subrostral process and basal prow (Fig. 5F).

Terminal crenulated anus, bounded by four, small indistinct lobes. No anal cirri (Fig. 5G).

Methyl blue staining pattern: staining of post-branchial region laterally (mid-dorsal region not stained) until chaetiger 8. Staining of 13 ventral shields. Abdominal staining light. No staining of dorsal side of neuropodial lappets.

**Figure 6.**
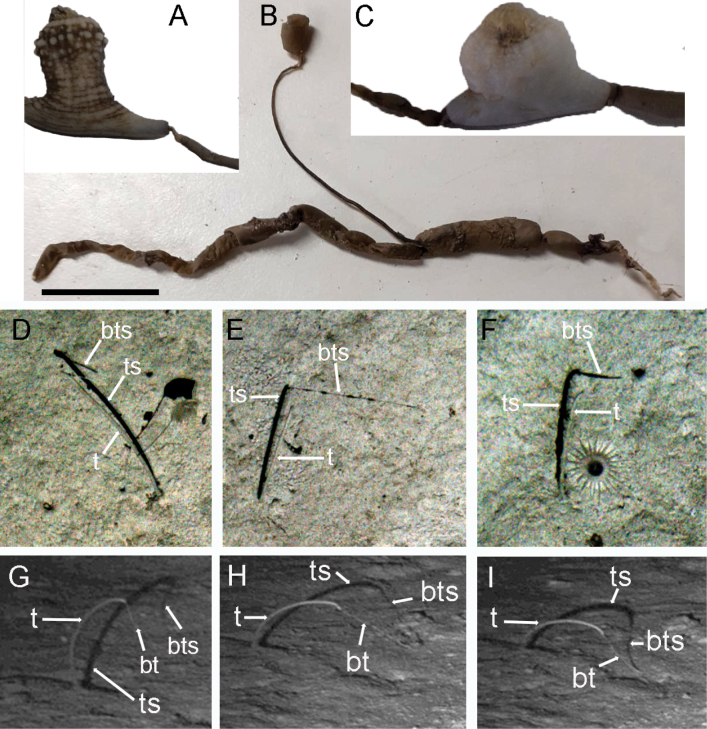
**A–C.***Melinnopsis nathanieli* sp. nov. association with **A.**Actiniaria, *Actinauge abyssorum*; **B.** Ascidian *Culeolus* sp.; **C.**Actiniaria, *Amphianthus bathybium*; **D–I.***Melinnopsis nathanieli* sp. inc.; **D–F.** Cropped images shot from Autosub5 mission 42 during RRS *James Cook* cruise 237 to PAP-SO (Huvenne 2024); **D.** Crop width 28.5 cm; **E.** Crop width 30.0 cm; **F.** Crop width 26.0 cm; **G–I.** Cropped images from Bathysnap time-lapse camera at PAP-SO station JC263-072. Abbreviations: bt = buccal tentacle, bts = buccal tentacle shadow, t = tube, ts = tube shadow. Scale bar: 2 cm (**B**).

Tube: fine-grained sediment tube with some foraminifera encrusted. The tube is lined with a thin, clear membrane. Length of tube ≤ 30 cm (Fig. 6B).

###### Ecology.

Found in association with the actiniarians *Actinauge abyssorum* Carlgren, 1934 and *Amphianthus bathybium* Hertwig, 1882, and the ascidian *Culeolus* sp. Herdman, 1881 (Fig. 6A, B, C). The worm tubes are grasped by the pedal disc of the Actiniaria (Fig. 6A, C).

###### Distribution.

Porcupine Abyssal Plain, northeast Atlantic, 4843–4850 m.

###### Etymology.

The new species is named for the second author’s son, Nathaniel Serpell-Stevens.

###### Remarks.

*Melinnopsis nathanieli* sp. nov. is morphologically very similar to *M. chadwicki* and *M. gardelli* from the eastern Australian margin. *Melinnopsis nathanieli* sp. nov. differs from these two species in having lateral wings that are slightly arched rather than highly arched, and possessing fewer abdominal uncini: only 16 in a row in *M. nathanieli* sp. nov. versus 30 in both *M. gardelli* and *M. chadwicki*. *Melinnopsis gardelli* also exhibits a ‘conspicuous stained band on the dorsal area when stained with methyl blue’ which was not observed in *M. nathanieli* sp. nov. Two other species of *Melinnopsis* have been described from the Atlantic (Table 4), *M. angolensis* and the type species, *M. atlantica*. *Melinnopsis nathanieli* sp. nov. differs from *M. angolensis* in lacking a dorsal serrated membrane on segment V (present in *M. angolensis*) and the possession of three teeth above the rostral tooth in the thoracic uncini (two teeth in *M. angolensis*).

**Table 4.** Comparison of all known species of *Melinnopsis* McIntosh, 1885. Updated table from Gunton et al. (2020: table 3), adapted from Hilbig (2005: table 3).* type species. TU, thoracic uncini. TC, thoracic chaetigers. Dashes (—) indicate not mentioned in original description.

Species	No. of TU	Dorsal membrane segment V	Buccal tentacles	Branchiae	No. of teeth above rostral tooth in TU	Body size (length: width)	Tube	Type locality
*M. abyssalis* (Hartman, 1969)	12	Absent	2 types: 1 large, many small	4 pairs: 1 anterior middle pair largest, 3 pairs in a crescent shape	3	52 mm: 3 mm	135–150 mm long, 3–4 mm wide, tapering slightly, smooth, dark silt	San Clemente basin, NE Pacific, 1920 m
*M. angolensis* Hilbig, 2005	13	Present: serrated (up to 21 teeth)	2 types: 4–6 large, 6 small	4 pairs: 1 anterior middle pair largest	2	21–50 mm: 3–4 mm	Up to 3 × length of worm, muddy with fine inner mucus lining	Angola Basin, SE Atlantic, 5385–5439 m
*M. annekovae* (Uschakov, 1952)	12	Absent: well-developed glandular band on segment four	2 types: 1 large > 7 mm, 3–4 small	4 pairs: 1 median pair largest	3 (5 teeth in one row)	—: 3 mm	Sturdy silted tube	Arctic Ocean, 51–1900 m
M. atlantica* McIntosh, 1885	14?	Present smooth (between segment 4 and 5)	None visible	4 pairs: unclear (no scars visible on re-examination of type material)	3 (top tooth indistinct) assuming pl. XXVIIA fig. 18 is thoracic	35 mm: 3 mm	Stiff cylinder, fine grey mud, Foraminifera attached	Off Chesapeake Bay, NW Atlantic, 3109 m
*M. arctica* (Annekova, 1931)	12	Present/indistinct , assuming 'dorsal fold' is the same as 'dorsal membrane'	2 types: 1 large, 2 small	4 pairs: anterior (inner) larger than others	2	25 mm: 3 mm	Solid tube like *Melinna cristata* covered in sand in the front part	Arctic Ocean, 165–480 m
*M. armipotens* (Moore, 1923)	13?	Absent/ indistinct	2 types: 1 large (12 mm long 8 mm wide), few small (1 mm long)	4 pairs: anterior (largest by one-third)	3	31 mm: 1.3 mm	—	Santa Catalina Islands, NE Pacific, 4070 m
*M. augeneri* Reuscher et al., 2015	13	Indistinct, no serration	2 types: 4 long thick and annulated, 3 small	4 pairs: arranged in continuous arch	2	14 mm: 0.8 mm	—	Goto-Kasayama Bank, west of Kyushu, 185 m
*M. chadwicki* Gunton et al., 2020	12	Absent/indistinct	2 types:1 long, 6 small	4 pairs: 1 pair slightly anterior	2	22 mm: 1 mm	Fine-grained sediment sometimes with green veins and Foraminifera	Eastern Australia, 1006–1257 m
*M. collaris* (Hartman, 1967)	12	Absent	2 types: 1 large, many small	4 pairs: crescent shape, 1 pair anterior	—	46–51 mm: 4.6 mm	Long, tough, covered with silt, internal membrane	Mid-Pacific Basin, 4041–4813 m
*M. dubita* (Hoagland, 1920)	12?	Indistinct ridge	2 types: 6 tentacles up to 15 mm, many smaller 3–7 mm	4 pairs: unclear	3 assuming pl. 51 fig. 5 thoracic	15 mm: 7 mm	Fine brown mud	Mindanao, Philippines, 920 m
*M. gardelli* Gunton et al., 2020	12	Indistinct	2 types: 1 long 34 mm and shorter tentacles 2 mm	4 pairs:1 pair anterior	3	40 mm: 4 mm	Fine-grained sediment sometimes with green veins and Foraminifera	Eastern Australia, 2520–2821 m
*M. mcintoshi* Reuscher, Fiege & Imajima, 2015	13	Present: smooth	2 types: 3 long thick, annulated, 4 thinner	4 pairs: 2 rows of 2	2	44 mm: 2 mm	—	Japan, Pacific Ocean, 164–5600 m
*M. monocera* (Augener, 1906)	12	Indistinct	2 types: 1 long (length 26 anterior segments), 6 short	4 pairs: unclear	2	28–42 mm: 2 mm	—	Caribbean, 212–310 m
*M. moorei* (Hartman, 1960) using Moore 1923	13? (17 TC)	Present: slightly serrated	—	4 pairs: cluster on each side	2	80 mm: —	Heavy mud walls	Off Santa Catalina and San Miguel Islands, NE Pacific, 495–3990 m
*Melinnopsis nathanieli* sp. nov.	12–14	Absent/ indistinct	2 types: 1 long up to 15 mm, 4 small 1 mm	4 pairs: 1 pair anterior	3	48 mm: 3 mm	Fine-grained sediment	Porcupine Abyssal Plain, northeast Atlantic, 4843–4850 m
*M. rostrata* (Wesenberg-Lund, 1950)	12	Present: 15–17 teeth	2 types: 5 long curled, 3 shorter	4 pairs: 1 pair anterior	—	72 mm: —	—	West of Greenland, Arctic, 3229 m
*M. shinkaiae* Jimi, Hookabe, Woo & Fujiwara 2025	12	Absent	2 types: 1 long, 5 small	4 pairs: 1 pair anterior	3	22 mm: 1 mm	mud, with sponge spicules	Daiichi-Kashima Seamount, Northwest Pacific, 3623 m
*M. somovi* (Uschakov, 1957)	12	Absent	2 types: 1 large, some small	3 pairs: Internal largest	2	15 mm: 1 mm	Silt with Foraminifera shells and small stones	Arctic, 1239–1694 m
*M. tentaculata* (Treadwell, 1906)	—	—	2 types: 1 large length of anterior region of body. 2 or 3 smaller	4 pairs: 2 rows, outer pair largest	3	9 mm: 1.5 mm	Thick mud tube with sponge spicules	Hawaii, 508–1358 m
*M. tetradentata* (Imajima, 2001)	13	Present: serrated 14 dentations	2 types: 1 long trihedral. Multiple shorter ones	4 pairs: 2 rows of 2	2	70 mm: 4 mm	Thick, dark, fine-grained mud particles, thin inner membrane	Tosa Bay, Japan, 400–800 m

*Melinnopsis nathanieli* sp. nov. differs from *M. atlantica* in lacking a dorsal membrane between segments IV and V (present in *M. atlantica*) and possessing a flexible tube composed of fine sediment with few attached foraminifera (cylindrical tube rigid and composed of greyish sediment with many foraminifera attached in *M. atlantica*). Furthermore, the depth distribution differs between the two species, *M. atlantica* was recovered from 1700 fms (3109 m), whereas *M. nathanieli* sp. nov. was collected from 4843–4850 m.

Since its description in 1885 there has only been a single additional report of *M. atlantica* (OBIS 2025), a specimen collected from the Golfe de Gascogne/ Bay of Biscay, northeast Atlantic in 1973 at 1180 m (Olu et al. 2025). It is unclear whether this specimen is *M. atlantica* or potentially another new species. We suggest that due to the differences in depth and geographical location, it is likely to be a different species and *M. atlantica* is not distributed in the northeast Atlantic

Unfortunately, our holotype specimen was in­complete with the end of the abdomen missing. Most of the specimens collected in 2023 and 2024, which were fixed in ethanol and suitable for genetic analysis, were in poor condition, often missing the posterior end. We selected the holotype based on condition of the specimen and availability of genetic data.

## ﻿Discussion

We describe a new species of *Melinnopsis*, *M. nathanieli* sp. nov., from abyssal depths in the northeast Atlantic. This is the first species of *Melinnopsis* to be described from the northeast Atlantic, with the nearest previously described species from the Angola Basin, southeast Atlantic (*M. angolensis*) and off Chesapeake Bay, northwest Atlantic (*M. atlantica*). *Melinnopsis nathanieli* sp. nov. displays genetic and morphological differences from all other described species of *Melinnopsis*.

*Melinnopsis nathanieli* sp. nov. is genetically very similar (interspecific COI pairwise distance 3.5%) to *M. gardelli* described from the eastern Australian margin at 2520–2821 m depth. This is lower than previous values for COI interspecific genetic difference suggested by Gunton et al. (2025) for the family Melinnidae (5.8–33.6%). Unfortunately, the Phylum Annelida lacks a universal barcoding gap, the separation between intraspecific and interspecific genetic distances. Indeed, the interspecific gap appears to differ between genera and families, for example, Syllidae (*Anguillosyllis*) (pairwise difference 6.6–15.1%) (Drennan et al. 2025), Goniadidae (pairwise difference 12.0–28.1%), *Spiophanes* (pairwise difference 4.0–14.4%) (Meißner et al. 2023), Onuphidae (p-distance 9.6–18.1%) (Budaeva et al. 2024) and the average for multiple families (K2P 16.5%) (Carr et al. 2011). Despite this high genetic similarity, given the difference in bathymetric range (*M. gardelli*: 2520–2821 m, *M. nathanieli* sp. nov. 4843–4850 m), widely separated type locality (ca 19,000 km from the northeast Atlantic to the southwest Pacific Fig. 1B) and morphological differences, we here describe *M. nathanieli* sp. nov. as a new species, distinct from *M. gardelli*.

We further propose that *M. gardelli* may not be a single species, but a complex of multiple species. In our phylogenetic analysis, *M. nathanieli* sp. nov. fell within a clade containing species attributed to *M. gardelli*, with a single *M. gardelli* specimen AM W.52539 recovered as sister to a clade containing the remaining *M. gardelli* (AM W.50735, AM W.51476) and *M. nathanieli* sp. nov. specimens. A recent study investigating the genetic connectivity using the COI barcoding gene fragment of three annelid species, including *M. gardelli*, along the eastern Australian margin, revealed that *M. gardelli* displayed strong genetic structuring (Gunton et al. 2025). Gunton et al. (2025) concluded that the intraspecific genetic differences were low enough to support *M. gardelli* as a single species; however, the differences suggesting strong genetic structuring may indicate incipient speciation. Following our molecular analysis using three genetic markers, we suggest that the records associated with the specimen AM W.52539 should be relabeled with the updated identification of *Melinnopsis* sp. Further detailed genetic analysis including additional markers for the specimens along the Australian margin are needed to resolve the *M. gardelli* species complex, which is beyond the scope of this study.

*Melinnopsis nathanieli* sp. nov. has an interesting association with some species of Actiniaria and Ascidiacea, in which the tube of the melinnid acts as a biogenic attachment substrate. One of the Actinaria species, *Actinauge abyssorum*, observed attached to melinnid tubes in this study has also been reported attached to the top of a dead sponge stalk, *Hyalonema* sp. in the abyssal northeast Pacific (Beaulieu 2001). It is likely both the sponge stalk and melinnid tube act as a vertical living space extending the associated ascidians into the benthic boundary layer flow thus improving the ascidians’ ability to suspension feed. It is unclear whether the host (sponge or worm) derives any benefit or harm from the relationship. This association between *Melinnopsis* and actinarians and ascidians has been noted since the start of the PAP-SO time series in the mid-1980s (AS-S unpublished data). Indeed, images of tubeworms with ascidians attached have been observed from Autosub5 Mission 42, Stn. JC237-053 to the Porcupine Abyssal plain (Fig. 6D) suggesting the association is not uncommon.

*Melinnopsis* tube worms are among the most frequently imaged polychaetes at PAP-SO (BJ Bett pers. comm. 13 October 2025). Images from a Bathysnap time-lapse camera at PAP-SO station JC263-072 deployed on 8 June 2024 and recovered 5 June 2025 included a tube worm in the camera frame (Fig. 6G–I). These images showed the worm tube positioned at an acute angle to the sediment, the tube moved position to become more upright (Fig. 6G) or bent towards the sediment surface (Fig. 6H, I). The buccal tentacle of the worm generally protruded out of the tube and was seen ‘midwater fishing’. There were also examples where the tentacle appeared to be in contact with the seabed (Fig. 6H, I) (A. Gates pers. comm. 15 October 2025). It is highly likely the Bathysnap and Autosub5 images (Fig. 6D–I) are of *M. nathanieli* sp. nov., given the distinctive tube which protrudes perpendicular to the sediment surface (also seen in seafloor images of the closely related *M. shinkaiae*, Jimi et al. 2025), and long buccal tentacle. Furthermore, *M. nathanieli* sp. nov. is the only *Melinnopsis* species retrieved in trawls from the PAP during JC237 and JC263. Since it is not possible to conclusively determine if the seafloor images are of *M. nathanieli* sp. nov. or another species, the image-based identifications are referred to as *Melinnopsis nathanieli* sp. inc. according to recommendations of Horton et al. (2021), to indicate ‘uncertain identification’. Precision sampling using a suction arm attached to an ROV would be needed to confirm the identification of suspected *Melinnopsis* specimens, followed by further morphological and molecular examination such as in the study of Jimi et al. (2025).

Although our new species agrees well with the current generic diagnosis of *Melinnopsis*, the generic diagnosis must be revised. The most recent diagnosis by Reuscher et al. (2015) included ‘large buccal tentacles occurring along with smaller ones’. However, a large buccal tentacle was not observed in our examination of the type species, *M. atlantica*, neither was this character mentioned in McIntosh’s original description (McIntosh 1885). Unfortunately, the holotype of *M. atlantica* is in poor condition, consisting of two short anterior fragments and one posterior fragment, thus preventing a redescription of the type species here. Presently, we cannot confirm the presence or absence of the large buccal tentacle in *M. atlantica*. Specimens from the type locality off Chesapeake Bay should be collected, a neotype designated, examined, and sequenced to redescribe the type species, revise the generic definition, and allow a subsequent revision of the entire genus.

## ﻿Conclusion

The present study describes a new species of abyssal tubicolous polychaete from the family Melinnidae, *M. nathanieli* sp. nov. This description will assist with species-level identification from seafloor imagery and act as a springboard for future publications on annelids from the Porcupine Abyssal Plain.

## Supplementary Material

XML Treatment for Melinnopsis

XML Treatment for Melinnopsis nathanieli

## ﻿Additional information

### Use of AI

No use of AI was reported.

### Author ORCIDs

Laetitia M. Gunton https://orcid.org/0000-0003-4758-4974

Amanda Serpell-Stevens https://orcid.org/0009-0009-1153-3033

Tammy Horton https://orcid.org/0000-0003-4250-1068

### Data availability

All of the data that support the findings of this study are available in the main text.
